# Analysis of the Free-Energy Surface of Proteins from Reversible Folding Simulations

**DOI:** 10.1371/journal.pcbi.1000428

**Published:** 2009-07-10

**Authors:** Lucy R. Allen, Sergei V. Krivov, Emanuele Paci

**Affiliations:** 1 School of Physics & Astronomy, University of Leeds, Leeds, United Kingdom; 2 Institute of Molecular and Cellular Biology, University of Leeds, Leeds, United Kingdom; University of North Carolina at Chapel Hill, United States of America

## Abstract

Computer generated trajectories can, in principle, reveal the folding pathways of a protein at atomic resolution and possibly suggest general and simple rules for predicting the folded structure of a given sequence. While such reversible folding trajectories can only be determined *ab initio* using all-atom transferable force-fields for a few small proteins, they can be determined for a large number of proteins using coarse-grained and structure-based force-fields, in which a known folded structure is by construction the absolute energy and free-energy minimum. Here we use a model of the fast folding helical *λ*-repressor protein to generate trajectories in which native and non-native states are in equilibrium and transitions are accurately sampled. Yet, representation of the free-energy surface, which underlies the thermodynamic and dynamic properties of the protein model, from such a trajectory remains a challenge. Projections over one or a small number of arbitrarily chosen progress variables often hide the most important features of such surfaces. The results unequivocally show that an unprojected representation of the free-energy surface provides important and unbiased information and allows a simple and meaningful description of many-dimensional, heterogeneous trajectories, providing new insight into the possible mechanisms of fast-folding proteins.

## Author Summary

The process of protein folding is a complex transition from a disordered to an ordered state. Here, we simulate a specific fast-folding protein at the point at which the native and denatured states are at equilibrium and show that obtaining an accurate description of the mechanisms of folding and unfolding is far from trivial. Using simple quantities which quantify the degree of native order is, in the case of this protein, clearly misleading. We show that an unbiased representation of the free-energy surface can be obtained; using such a representation we are able to redesign the landscape and thus modify, upon site-specific “mutations”, the folding and unfolding rates. This leads us to formulate a hypothesis to explain the very fast folding of many proteins.

## Introduction

It is commonly believed that, with sufficient computer time and accurate models, the energy landscape of any protein could be mapped out from its sequence by running and analysing folding simulations, thus making possible prediction of both folding mechanism and native structure. This is not yet possible: folding events have only been observed in simulations of very small, fast (sub *µ*s) folders [1],[2]. The main reason for this limitation is the computational expense of accurate protein models, which typically allow only a few ns of dynamics to be generated within a reasonable timescale of weeks or months. Another obstacle may be the models themselves, whose accuracy is difficult to assess for the very same reason. Nevertheless, with the development of faster processors, new sampling techniques and improved force-fields, equilibrium simulations of accurate protein models are likely to become achievable in a not-too-distant future. The analysis of such equilibrium simulations, however, poses another problem. Determining and representing the free-energy surface, which underlies the thermodynamic and dynamic properties of the model, from an equilibrium simulation in a meaningful way is a complicated task, and numerous studies have been devoted to this task [3]–[10]. Most commonly, the free energy surface has been projected on a small number (usually one or two) progress variables, such as the root mean square distance (RMSD) from the native structure, the radius of gyration R_g_ or the number of native contacts. Integrating over all other degrees of freedom induces a free energy landscape as a function of these coordinates, which typically exhibits a maximum (the transition state) at some point between the minima representing the ensembles of denatured states and the native state. This enormous projection is highly problematic, as features inherent to the multi-dimensional nature of the true folding space, such as the presence of local minima, can be lost. Most importantly, the existence and height of free-energy barriers in these projections are often inaccurate. One solution to this problem is provided by a recently proposed method to determine and represent unprojected free-energy surfaces [11],[12]. Based on disconnectivity graphs [13], the method aims to group conformations into free-energy minima not using geometrical criteria but equilibrium dynamics. More recently this method has been extended to determine a one-dimensional projected free-energy surface in terms of a reaction coordinate that preserves the free energy barrier, and the coordinate dependent diffusion coefficient [14]. This method has previously been applied to model systems such as a 20-residue designed peptide that folds to a double hairpin [10] and a coarse-grained model of a protein under mechanical force [15].

The problem of how best to analyse an equilibrium folding trajectory cannot be addressed with detailed models for the reasons mentioned above. Reversible folding trajectories can, however, be obtained with structure based models, hence their broad popularity in computational folding studies [16]–[25]. Using these models a sequence can fold from a random extended conformation to the native structure, reach equilibrium and unfold and refold a large number of times in a typical trajectory. Depending on the target structure, the free-energy barrier for unfolding may still be exceedingly large and folding too slow to be observed. Such models disfavour non-native interactions, and are therefore strongly biased towards native interactions. Consequently their accuracy in describing the folding behaviour of real proteins has been debated [26],[27]. Nevertheless they predict features which are believed to be characteristic of the folding landscapes of real proteins, such as the presence of intermediates [28]–[30] and downhill folding [31]–[36], and are undoubtedly useful for understanding the general features of landscapes. Structure based models are also easily malleable and sensitive to individual interactions [37]–[39], allowing the effects of perturbations of the free energy landscape to be investigated.

**Figure 1:**
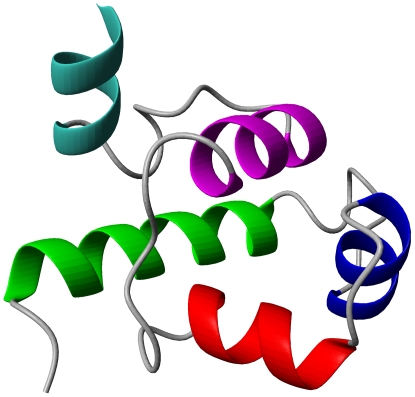
Experimental native structure of *λ*-repressor (1lmb). Helices occur in positions 9–23, 33–39, 44–51, 61–69 and 79–85.

In this paper we use both geometric projections and the unprojected representation described above to extract free energy surfaces from reversible folding simulations. The specific landscape which is probed is that of a 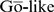 model of the N-terminal domain of phage *λ*-repressor protein [40] at its melting temperature. We chose this five-helix bundle protein (Figure 1) because it has been extensively studied experimentally [41]–[49], and has been shown to be a very fast (∼3600 s^−1^ at 37°C and 0 M urea), two-state folder [40]. The two analyses are compared, and states which are hidden by the geometric projection are discovered. In particular, hidden parallel pathways and intermediates are found to play an important role in the fast folding of the model. Removing these features by perturbing the model results in a more than two-fold reduction in the folding rate. The aim of this work is not to discuss the merits of structure-based models for reproducing known experimental properties of proteins, but rather to demonstrate the importance of a thorough analysis of equilibrium kinetics which is not biased by the choice of arbitrary projection variables.

## Methods

### Simulations

Simulations of *λ*-repressor and two variants have been performed using the force-field of Karanicolas and Brooks [50],[51] implemented in the program CHARMM [52]. In this structure-based C*_α_* model, interactions are attractive if they are present in the experimental native state and repulsive otherwise. The magnitude and range of the interactions depend on the chemical properties of the residues and their separation in the experimental structure. The dihedral part of the potential is sequence-specific.

The force-field was modified to generate two variants, A and B. In variant A, the magnitudes of the non-bonded interactions between residue 73 and residues 80, 81 and 84 were increased by factors 1.75, 2.5 and 1.75, respectively. In variant B, attractive non-bonded interactions were introduced between residues 43 and 48, and residues 44 and 47.

To maintain a constant temperature, Langevin dynamic simulations were performed with a timestep of 15 fs and a uniform friction coefficient of 1 ps^−1^ acting on all particles. We verified that the friction coefficient corresponds to the regime in which rates are proportional to the friction coefficient, i.e., we use a friction low enough to guarantee the generation of a large sample of folding/unfolding events, but which is not in a ballistic, low friction regime [53].

Simulations of each protein were performed over a broad range of temperatures, and the Weighted Histogram Analysis Method (WHAM) [54] used to calculate specific heat capacity curves. The temperature at which the specific heat reached a maximum was identified as the melting temperature 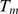. Longer (30 µs) simulations were run at this temperature, with coordinates being saved every 7.5 ps. More than 600 folding events were observed for the wild-type protein.

### Analysis

The equilibrium trajectories are first analysed by projection onto the geometric coordinates RMSD from the native structure and fraction of native contacts formed (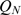). Contacts are considered to be present if two C*_α_* atoms are separated in sequence by more than 4 residues and are less than 12 Å apart, and the native contact map is constructed from the experimentally determined native structure.

The further analysis consists of three stages. First, the trajectory is used to build a network, the equilibrium kinetic network (EKN), which describes the system kinetics at equilibrium. This is obtained by clustering the trajectory in the principal component space defined by the distance between selected atom pairs, and counting the number of transitions between clusters (see Text S1 for details).

Once such network has been determined, its free energy profile (FEP) is built using a procedure which is described in detail elsewhere [14],[55] and in Text S1. The FEP is plotted as a function of a “natural coordinate” which is constructed so that the diffusion coefficient is constant along the profile, and the mean first passage times (MFPTs) between any two points can be calculated using Kramer's equation [10]. For sequential folding pathways, the heights of the barriers on the FEP of the system are exact. If parallel pathways are present, however, usually only the highest barrier is exact. To overcome this problem, any two states can be chosen and the FEP between only these two states built, giving an exact barrier height. The third stage of the process is to use the FEP to iteratively partition the network into basins to generate a simplified EKN (SEKN) which describes the system kinetics. The procedure by which the SEKN is generated is described below.

The simplified equilibrium kinetic network (SEKN), which describes the inter-basin kinetics, is constructed by iteratively partitioning the EKN into basins. To do this, notable barriers are first identified in the FEP. Two representative nodes on either side of the barrier are selected in the EKN, and the network divided by computing the “minimum cut” [11],[12] between these two nodes. This procedure is applied iteratively until there are no notable internal barriers in any of the basins. The number of effective transitions between each pair of directly connected basins is then computed by assuming diffusive dynamics and using Kramers' equation to estimate the mean first passage time from one basin to the other [55].

For all the analyses shown below, we assessed the convergence by repeating the analysis for the first and second half of the trajectories. The networks are in all cases identical and the populations of basins differ at most by 10% (see Text S1 for details).

## Results

### “Geometric” analysis

**Figure 2:**
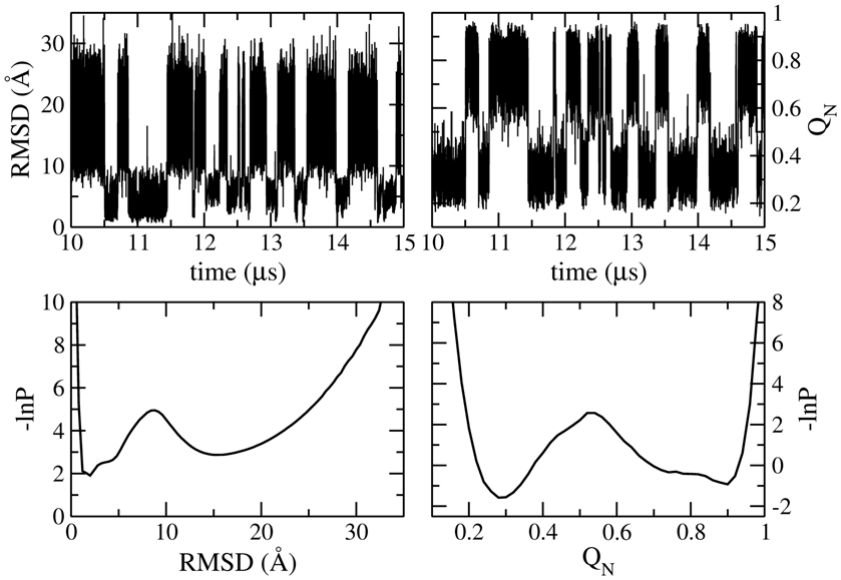
Projection of equilibrium trajectory onto geometric progression variables. (A) Timeseries' of RMSD from experimental structure and fraction of native contacts Q*_N_* from simulation at 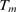. (B) Potential of mean force as a function of RMSD and Q*_N_*.

**Figure 3:**
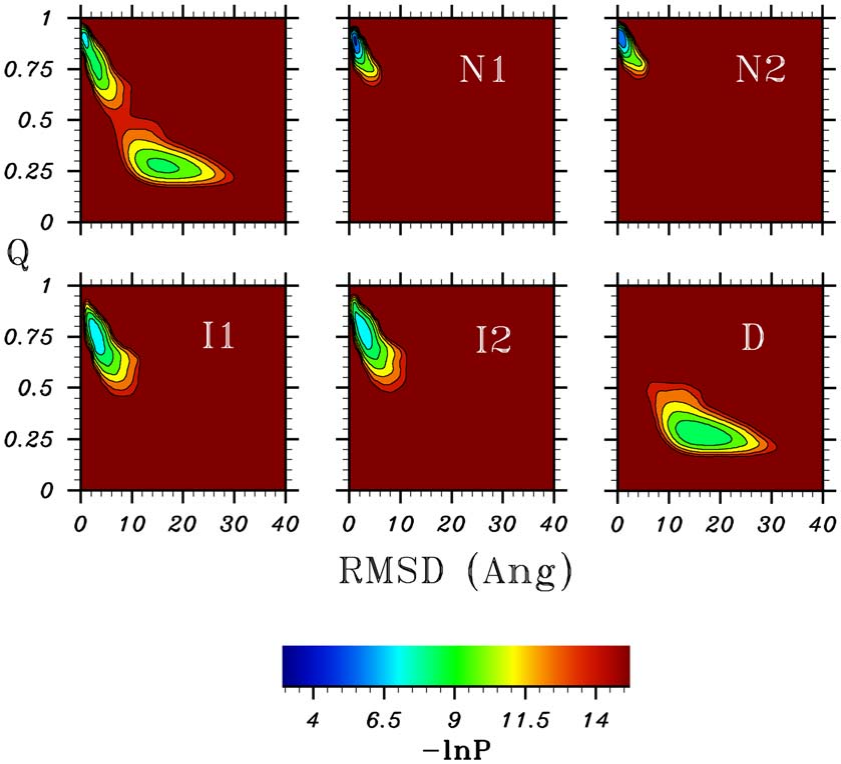
Projections of the complete trajectory, and of the trajectory split into the five different states from the SEKN, onto Q*_N_*/RMSD.

At first glance, the folding behaviour of the structure-based model of *λ*-repressor appears to be two state. The specific heat profile shows a sharp peak at the melting temperature (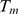), indicating highly cooperative folding behaviour. Timeseries' of geometric coordinates such as the number of native contacts Q*_N_* and RMSD (shown in Figure 2A) switch rapidly between two states: one is characterised by high Q*_N_* and low RMSD (i.e. a native-like state) and the other by low Q*_N_* and high RMSD (a denatured-like state). According to these coordinates, therefore, folding of the model is a two-state process. More than 600 folding events occur within the simulation time of 30 *µ*s. Figure 2B shows free energy profiles built from projections of the trajectories onto the two coordinates. Clearly two stable states are present, separated by a small barrier. The relative stabilities of the two states, however, differ according to the coordinate used: while on the RMSD projection the native state is marginally more stable than the denatured state, the opposite is true when Q*_N_* is used as the reaction coordinate. The size of the barrier for the folding transition also differs from 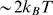 in the RMSD projection to 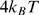 in the Q*_N_* projection. These differences highlight the difficulties involved in analysing trajectories by projecting them onto single geometric reaction coordinates. A better solution may be to project onto a plane defined by several reaction coordinates: the top left panel of Figure 3 shows a projection of the trajectory at 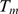 onto both RMSD and Q*_N_*. This projection appears to be more reliable, with the two states being clearly separated, and an energy barrier of around 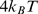. However, as we will show in the next section, even this projection hides detail which is important in understanding the folding process.

### Unprojected analysis

**Figure 4:**
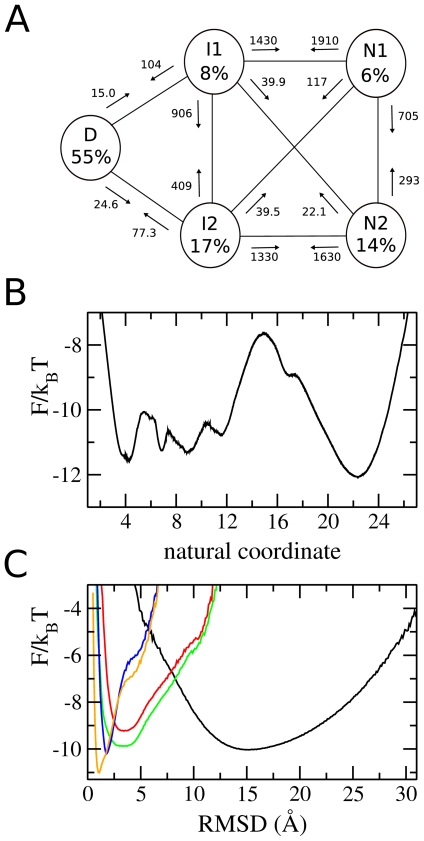
Results of detailed analysis of *λ*-repressor equilibrium simulations. (A) simplified equilibrium kinetic network (SEKN). Rates of exchanges between states are shown in *µ*s^−1^. (B) unprojected free energy (FEP). C: FEP of each basin as a function of RMSD.

Figure 4 shows the results of the more detailed analysis of the trajectory at 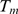. Panel B shows the free energy profile (FEP) as a function of the “natural coordinate” described previously: five stable states are identifiable. These five free energy basins are plotted as a function of RMSD in panel C. At low values of RMSD (∼2 Å), two native basins are present, labelled **n1** and **n2**. Two intermediate states **i1** and **i2** lie at slightly higher RMSD (∼4 Å). The denatured state **d** is a broad basin with a minimum at RMSD ∼15 Å. Figure 3 shows the positions of the five states on a projection onto the two-dimensional reaction coordinate (RMSD, Q*_N_*): the two native and two intermediate states overlap considerably, making them indistinguishable in the overall projection. The SEKN, which provides information about the populations and kinetics of the network, is shown in of Figure 4A. Two parallel pathways can be identified as the main folding routes: **d**→**i1→n1** and **d→i2→n2**. Folding also occurs through **i1→n2** and **i2→n1**, but at a much slower rate. Interchange between the two native states (**n1→n2**) and between the two intermediate states (**i1→i2**) is rapid, suggesting that they are separated only by small free energy barriers. From FEPs plotted between the states the size of these barriers can be estimated as 3 and 2.5 k*_B_*T for the native and intermediate states respectively. Exchange between the native and intermediate states (i.e. **n1**→**i1** and **n2→i2**), is also fast, and these states are separated by energy barriers of only ∼2 k*_B_*T. The rate limiting step in folding is the transition between the denatured and intermediate states, for which the energy barrier is ∼5 k*_B_*T.

**Figure 5:**
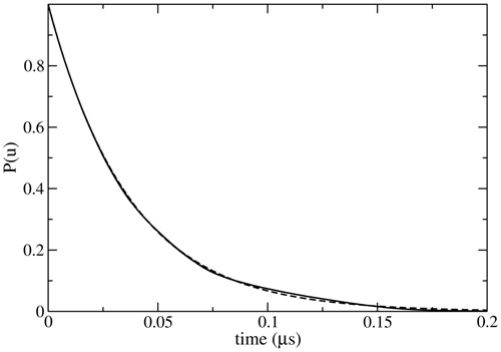
“Survival” probability of the denatured state [63] i.e., the probability that an unfolded conformation remains non-native. The dotted line shows a single exponential fit to the distribution, with 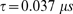.

The distribution of folding times from **d** to **n1**/**n2** is shown in Figure 5. The curve fits a single exponential distribution: the equilibration of the native and intermediate states is sufficiently fast compared to the **d** to **i1**/**i2** step that a single time constant can be used to describe the folding with reasonable accuracy. This has the consequence that, should the folding pathways described above be representative of the real protein, a kinetic experiment would not reveal the presence of the intermediate state, or indeed the parallel pathways.

### Structure of the intermediate states

**Figure 6:**
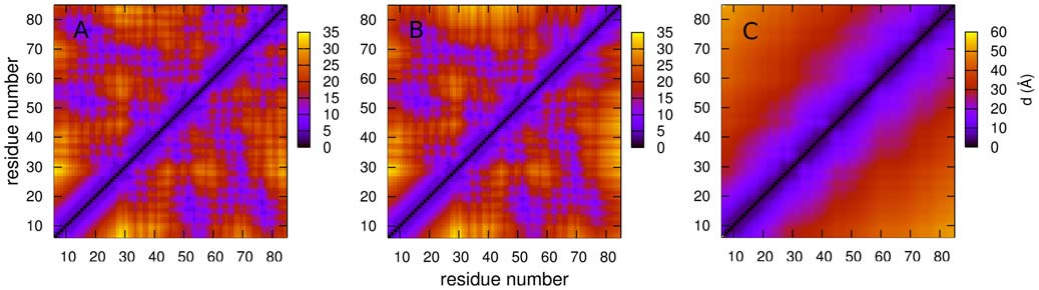
Maps showing average pairwise residue distances in the n1 (A), i1 (B) and d (C) states.

**Figure 7:**
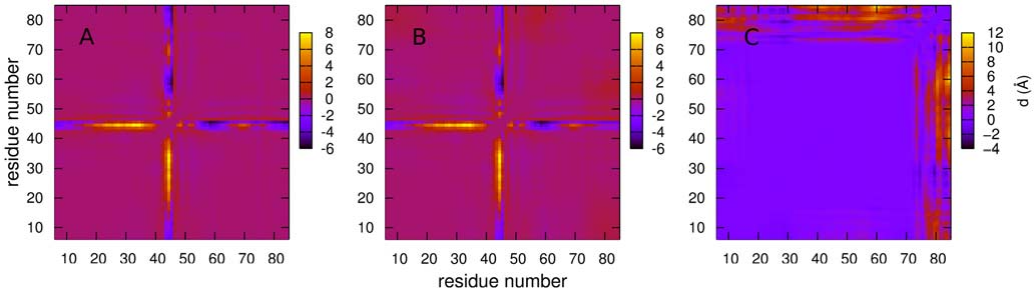
Maps showing changes in average pairwise residue distances between the n1 and n2 states (A), i1 and i2 states (B) and n2 and i2 states (C).

**Figure 8:**
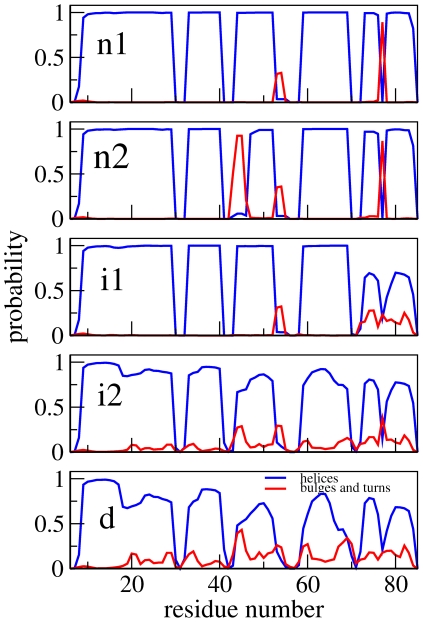
Secondary structure propensity of the different states, calculated using DSSP [64].

**Figure 9:**
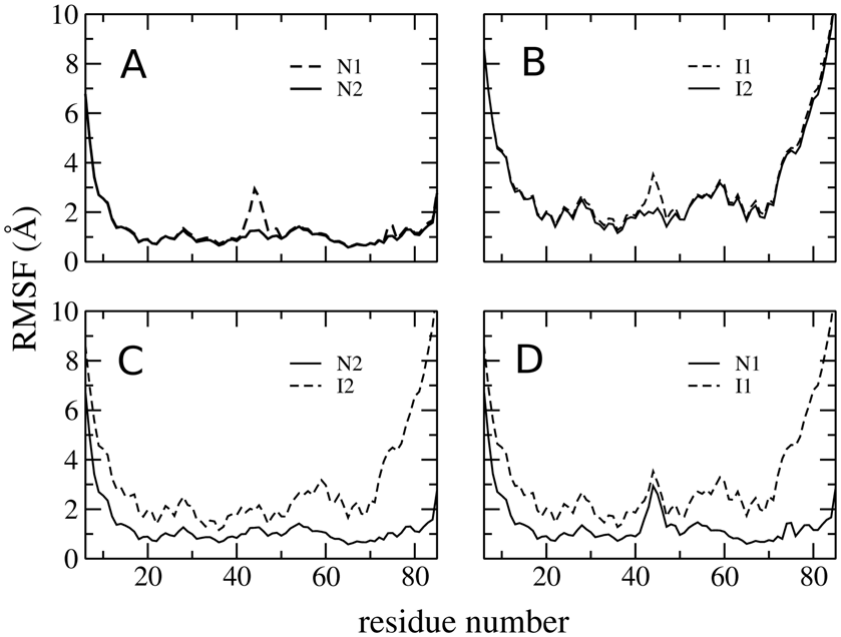
RMSFs for each residue in the native and intermediate states.

**Figure 10:**
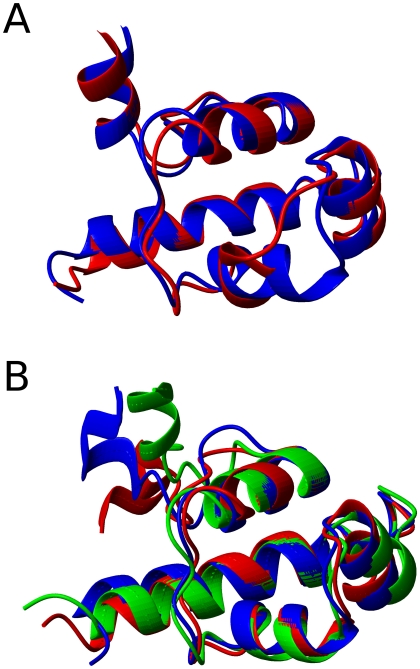
Representative structures (most populated cluster centres) of states identified in unbiased analysis of equilibrium trajectory. (A) States n1 (in blue) and n2 (in red). (B) State i.

Panels A and B of Figure 6 show matrices of average inter-residue distances for the **n1** and **i1** states. The two are similar, with local contacts being present in the helical regions (residues 9–23, 33–39, 44–51, 61–69 and 79–85), as well as several regions of non-local contacts. The differences between the two states lie in the helix 5 region, in which the non-local contacts are significantly reduced. This can be more clearly seen in the matrix of differences between the pairwise distances (Figure 7C): helix 5 moves away from the rest of the protein during the transition from state **n1** to state **i1**. The distance matrices for the **n2** and **i2** states, which are not shown, reveal an analogous change. The secondary structure propensities for the native and intermediate states are shown in Figure 8. Whilst all five helices are always present in the two native states, the helicity, and particularly the helicity of helix 5, is slightly diminished in the intermediate states: in both **i1** and **i2** helix 5 is only present in around 75% of structures. The positional root mean fluctuations (RMSF) of each residue (Figure 9) for the intermediate and native states also indicate that the largest differences are in the helix 5 region, in which the flexibility is significantly larger in the intermediate states than in the native states. Analysis of contact probabilities reveals that 12 attractive native contacts are lost (or present in at least 50% fewer structures) in the transitions from **n1** to **i1** (or **n2** to **i2**), and these are all made by residues in helix 5 and the loop between helices 4 and 5. Together these analyses give a clear picture of the two intermediate substates. In state **i2** helices 1–4 are native-like, and helix 5 is generally formed but detached from the rest of the structure. State **i1** is similar, but with a slightly frayed helices 1–4. Figure 10B shows representative structures of the **i2** state.

As non-native interactions are not included in the model, entropy must play an important part in stabilising the intermediate states. In this case the loss of enthalpy that results from breaking the long-range native contacts made by helix 5 is balanced by the increased entropy associated with the freedom of the helix.

### Origin of the splitting of the native and intermediate states

The differences between the **n1** and **n2** states and between the **i1** and **i2** states are more subtle. The left-hand panel of Figure 7 shows the changes in average pairwise distances between the two native states; the differences are very clearly localised in the region of residues 42–47 (part of the loop region between helices 2 and 3, and the N terminal of helix 3). This difference can also been seen in the secondary structure propensities of the two states (Figure 8): helix 3 is slightly shorter in state **n1**, commencing at residue 47 rather than residue 44. The region between the two helices, which has no secondary structure elements in state **n2**, is classified as a bulge or a turn in state **n1**.

Figure 9A shows the RMSF for each residue in the two native states (**n1** and **n2**). Again, the differences are localised in the same area, with state **n1** being more flexible in this region than **n2**. The increased entropy associated with the increased flexibility in state **n1** is compensated for by a loss of attractive contacts: Table 1 shows that several attractive contact probabilities, all in the residue 42–47 region, are significantly reduced in **n1** compared to **n2**. Figure 10A shows representative structures of states **n1** and **n2**.

**Table 1:** List of attractive contacts whose probability differs by more than 0.2 between **n1** and **n2**.

i	j	% in n1	% in n2
33	44	0	20
34	44	0	29
36	47	46	66
37	42	33	79
42	47	40	78
42	50	58	83

These analyses show that the two native states arise from a careful balance of enthalpy and entropy: whilst **n1** loses out in enthalpic terms by having fewer attractive contacts than **n2**, it gains entropy from increased flexibility of the loop. This is also the case for the two intermediate states: again the changes are localised to the same loop region (Figure 7B), and the increased entropy associated by the flexibility of the loop in **i1** (Figure 9B) is balanced by a loss of contacts in this region (Table 2).

**Table 2:** List of attractive contacts whose probability differs by more than 0.2 between **i1** and **i2**.

i	j	% in i1	% in i2
33	44	0	26
33	47	41	61
34	44	0	43
37	42	40	84
42	47	56	89
42	50	56	81

### The denatured state

Figure 3 shows that the denatured state identified by the unprojected analysis is very similar in terms of RSMD and Q*_N_* to the denatured state identified by projection onto these coordinates. The enthalpic destabilization and high heterogeneity of the denatured state make it intrinsically difficult to study, both in experiment and simulation, and it is therefore interesting to characterize it to some extent here. As stated previously, the aim of this paper is not to reproduce the experimental properties of *λ*-repressor, or to debate the accuracy of coarse grained models. Nevertheless, it is a valuable exercise to make some comparison with experiment, as such a comparison could point in directions in which the model could be improved. The average radius of gyration of the denatured state in the simulation is 20.5 Å; this compares well with the value determined experimentally for a mutant of the same protein of 23±2 Å [44]. Both the experimental and simulation values of R_g_ are smaller than the value (26 Å) expected for a random coil [56], indicating that there are residual interactions in the denatured state. Certainly this is the case in the simulation: the average pairwise distance matrix for the denatured state (Figure 6) shows that although no long range interactions are present, a number of local contacts are formed, indicating the presence of some secondary structure. This can also be observed in the secondary structure propensity of this state (Figure 8): whilst the helices are diminished in this state, all five are present to some extent. Evidence of secondary structure in the denatured state has been found for a number of proteins [57],[58]. In fact, a recent NMR study of a mutant of *λ*-repressor in which the denatured state is populated under non-denaturing conditions showed that significant helical structure was present [48],[49]. In contrast to the simulation results presented here, however, the helicity was limited to the N-terminal region of the protein. This disagreement indicates that the high helicity observed in the simulation may well be an artifact of the model.

### Rational modification of the free energy surface

The malleability of the Go-like model, together with the above information about the folding mechanism, allow modifications of the model which alter the folding pathway. Such modifications are useful as, by comparing the folding rates of the wild-type and modified proteins, it may be possible to identify those features in the folding landscape of the wild-type which make it a fast folder. Here, two modifications have been made: one which removes the intermediate states from the pathway, and another which removes the parallel pathways.

**Figure 11:**
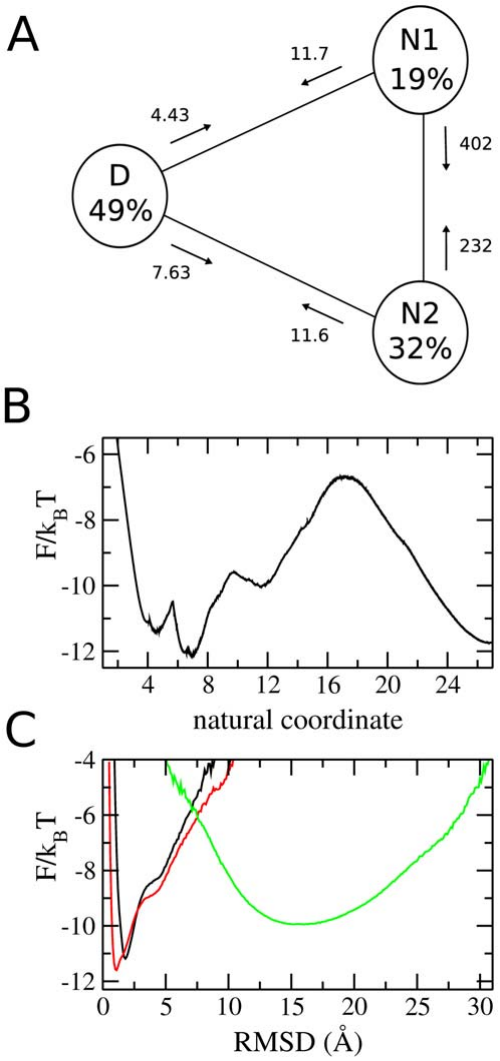
Results of detailed analysis of equilibrium simulation of model A. (A) the simplified equilibrium kinetic network (SEKN) shows three states, two rapidly interconverting native states, and the denatured state. Rates of exchanges between states are shown in *µ*s^−1^. (B) unprojected free energy (FEP). The intermediate state is no longer significantly populated, but still remains as roughness on the energy landscape. (C) FEP of each basin as a function of RMSD.

The first modification (A) was designed destabilize the intermediate state: the interactions of residue 73 with residues 80, 81 and 84 are strengthened. This should clamp helix 5 into its native position, and thus destabilise the intermediate state, in which helix 5 is not docked. The melting temperature of the modified model is slightly higher than the wild-type (327 K compared to 323 K) i.e., the modification marginally stabilises the native state. The FEP (Figure 11B) calculated from simulations at 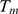 shows only three stable states; from the RMSD plot (Figure 11C) they can be identified as two native substates (**n1** and **n2**), and the denatured state. The intermediate states have been destabilized sufficiently that they are no longer significantly populated. Interchange between the native substates is rapid (see SEKN, Figure 11A SEKN), but the barrier between **n1**/**n2** and **d** is rarely crossed.

**Figure 12:**
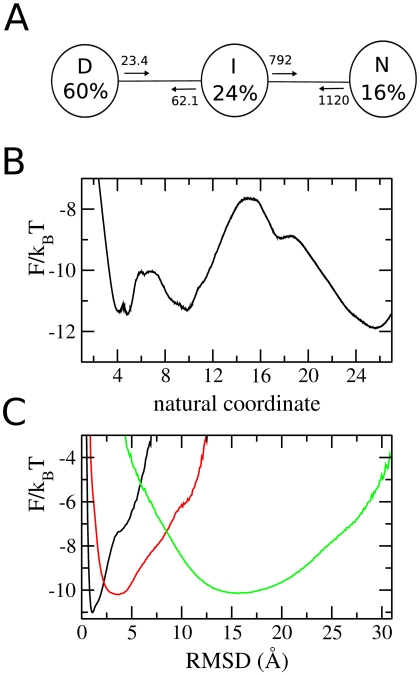
Results of detailed analysis of equilibrium simulation of model B. (A) the simplified equilibrium kinetic network (SEKN) shows three states, native, intermediate and denatured state. Folding occurs mainly through the pathway 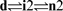. Rates of exchanges between states are shown in µs^−1^. (B) unprojected free energy (FEP) landscape. (C) FEP of each basin as a function of RMSD.

The second modification (B) was designed to force the model to fold via a single, rather than parallel, pathway. The above analysis shows that the native and intermediate substates differ mainly in the region of residues 42–47. Introducing attractive interactions between those pairs of residues which form contacts in state **n2** but not in **n1** should stabilise **n2** relative to **n1** and thus channel the flux into a single pathway. Two interactions were introduced in the design of model B: between residues 43 and 48, and 44 and 47. The SEKN for this model (Figure 12A) shows that the design was successful: the protein now folds via the pathway 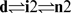.

Folding rates for the wild-type and two modified proteins, taken from the SEKN, are shown in Table 3. Folding rates are for the **d** to **i1**/**i2** transition for the wild-type and model B, as this is the rate limiting step, and for the **d** to **n1/n2** transition for model A. Both models fold significantly more slowly than the wild-type. This result is important as it shows that both the intermediates and parallel pathways are at least partially responsible for the observed fast folding of the wild-type model.

**Table 3:** Folding rates of *λ*-repressor and its mutants.

Protein	T (K)	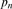	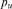	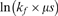	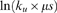
WT	323	0.464	0.535	3.68	5.20
Mutant A	327	0.511	0.489	2.49	3.15
Mutant B	327	0.399	0.601	3.15	4.13

## Discussion

In this paper we have investigated several ways of analysing equilibrium simulations: traditional geometric analysis, in which the trajectory is projected onto one or several reaction-coordinates, and a recently proposed method which uses an unprojected representation of the free energy landscape. In particular we have focused on the folding of a structure-based model of a small, fast-folding five-helix bundle, *λ*-repressor, which has been widely studied experimentally. Fluorescence and NMR measurements indicate that *λ*-repressor is a two state folder which can be transformed into a barrierless folder via specific mutations. The simulations agree with experiment when analysed using RMSD and Q*_N_* as reaction coordinates: the model appears to fold quickly via a two state transition. The unprojected analysis, however, reveals more complexity: an obligatory intermediate state is present in the pathway, and the native and intermediate states are split into two “sub-states”. The intermediate states, which cannot be distinguished from the native states in projections over conventional geometrical coordinates, are stabilised by a balance of enthalpy and entropy: helices 1–4 are natively docked and helix 5 is generally formed but detached.

The characterisation of the different states on the folding pathway revealed by the detailed analysis allowed the design of “mutants” of the model which fold via different mechanisms. In one mutant, the intermediate states were destabilised so that they were no longer populated i.e., folding occurred directly from the denatured state to the two native substates. The role of intermediates in folding has been widely debated: it appears that, depending on their stability [59] they may act as kinetic traps and thus slow folding [60], or as an important stepping stone, channeling flux to the native state and thus accelerating folding [61],[62]. The analysis of the folding of both the “wild-type” model and the “mutant” showed that the rate of folding was significantly smaller for the mutant. This indicates that, for our model, the intermediate state guides the protein towards the native state, thus accelerating folding. Another mutant was designed to fold via a single pathway i.e., the native and intermediate substates of one pathway were stabilized so that the other pathway was no longer significantly populated. The resulting folding rates were smaller than the wild-type, and approximately equal to the rate that could be predicted from considering only one path of the wild-type. This result demonstrates that, at least for this model of *λ*-repressor, the fast observed folding rates are at least partially due to the presence of parallel pathways.

It is well known that experimental probes of protein folding are often localised and therefore may not be sensitive to structural changes in distant parts of the protein. In this paper we have shown that an analogous problem exists in simulation: the projection of reversible trajectories onto geometric reaction coordinates can hide important features of the folding pathway. Such features can, however, be uncovered by a more detailed analysis such as the unprojected representation used here. This detailed analysis reveals important characteristics of the folding landscape of a structure-based model of a fast-folding protein which help to explain how it folds so quickly.

## Supporting Information

Text S1 Supporting Information
(0.16 MB PDF)
