# Supplementary material for: Analysis of the Free-Energy Surface of Proteins from Reversible Folding Simulations
Source: PLoS Comput Biol. 2009 Jul 10;5(7):e1000428. doi: 10.1371/journal.pcbi.1000428 (PMC2700257; doi:10.1371/journal.pcbi.1000428)
Supplement: Text S1 — Supporting Information (0.16 MB PDF) [file pcbi.1000428.s001.pdf]

Clustering procedure

The equilibrium kinetic network (EKN) is an undirected capacitated network which describes the equilibrium kinetics of the system (1,2). The EKN is obtained by clustering the trajectory into nodes which contain sets of similar structures.

To reduce the dimensionality of the system before the clustering procedure is applied we use bond principal component analysis (BPCA). BPCA is performed with selected atom pairs only to reduce computational expense further as well as to increase the signal to noise ratio (see Reference (3) for a similar approach, where all atom pairs are used). To select atom pairs for BPCA, the potential of mean force associated with their distance is first determined for each pair in the system. Examples of two such profiles are shown in Figure 1. Atom pairs which give a profile with more than one free energy basin (the right-hand panel in Figure 1, for example) are included in the PCA, and all others are discarded (the left-hand panel in Figure 1). BPCA is performed by calculation of an $N \times N$ covariance matrix, where N is the number of selected atom pairs:

$$C_{ij} = \langle (r_{p_i}(t) - \bar{r}_{p_i})(r_{p_j}(t) - \bar{r}_{p_j}) \rangle \tag{1}$$

where i and j are the labels of the pairs, r_{p_i} is the distance between the two atoms in pair p_i at time t and $\bar{r}$ represents the corresponding mean value. The eigenvectors corresponding to the three largest eigenvalues of C_{ij} (i.e., the first three principle components) are taken to be the basis vectors for the three-dimensional space in which clustering is performed. This "PCA space" is divided into cubes of equal size, each corresponding to a cluster. The size of the cubes are chosen such that there are 20000 clusters in total (this number is chosen to be as large as possible, but with the requirement that clusters are sufficiently populated to be statistically representative and not too large for numerical problems due to the diagonalisation of rate matrix). Each frame of the trajectory is assigned to a cluster according to its position in "PCA space", and the number of transitions (n_{ij}) between each pair of nodes (i and j) are counted. To impose detailed balance (which holds in the limit of infinitely large statistics) the capacities in the EKN are taken as $c_{ij} = (n_{ij} + n_{ji})/2$.

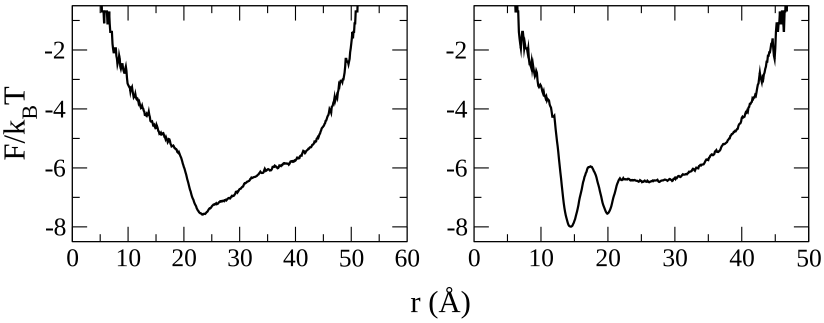

Figure 1: Demonstration of selection of atom pairs for bond PCA. The free energy profiles show $-\ln p(r) = F/k_BT$, where r is the bond distance, for two different atom pairs. The left-hand profile shows only a single basin: this atom pair (C_α^2-C_α^{28}) would not be included in the bond PCA. The right-hand profile shows two basins: this pair (C_α^{17}-C_α^{39}) would be included in the bond PCA.

Building the FEP

The free energy profiles for the EKN are constructed using a procedure (4, 5) which employs folding probability (6) (pfold) as the reaction coordinate. Folding probability was originally proposed as a reaction coordinate to separate folded and unfolded states in protein folding, but can be used as a reaction coordinate to separate any two regions of the configuration space. To compute the folding probability for each node of the EKN one has to specify two nodes A and B; these are usually taken to be the most populated nodes of two basins of interest. When no information about the basins is available it is convenient to take the most populated node (which is likely to represent the native state) as node A. To avoid choosing a node in the same basin as node A, a node B is created outside the network (4). Node B is connected with every node (i) in the network with capacity $c_{Bi} = \lambda N_i$, where $N_i = \sum_j n_{ji}$ is the population of node i, and λ is taken to be 10^{-4}. p^{fold} can then be determined for each node by solving $p_i^{fold} = \sum_j p_{ji} p_j^{fold}$ with the boundary conditions that $p_A^{fold} = 1$ and $p_B^{fold} = 0$, where p_{ji} are the components of the transition probability matrix obtained from the EKN (i.e., $p_{ji} = c_{ji} / \sum_k c_{ki}$). The nodes are then sorted according to their values of p^{fold}, which all lie in the range 0 to 1. Next, the FEP is built by considering a set of points, p_c between 0 and 1, and using them to "cut" the network into a set with $p^{fold} < p_c$ (set A) and a set with $p^{fold} > p_c$ (set B). For each value of p_c a point of the profile (Z_A, $-kT \ln(Z_{AB})$) is computed, where Z_A is the population of set A and Z_{AB} is the number of transitions between sets A and B. It is convenient to transform the Z_A coordinate into a coordinate (labelled "natural coordinate") where the diffusion coefficient is constant; this is done by numerically integrating the equation $dx = (2/\sqrt{\pi}) dZ_A / Z_{AB}$ (7).

Convergence of the free-energy representations

To confirm that sampling is sufficient and that the free-energy surface represented either as one-dimensional projection over the "natural coordinate" or as an equilibrium kinetic network is converged we separated the trajectory of the wild-type protein model into two halves, and performed the analysis on each half separately. The resulting FEPs are shown in Figure 2. As mentioned in the Analysis section, if parallel pathways are present then only the height of the largest barrier is meaningful. Figure 2 clearly shows that there is excellent agreement between the two halves of the trajectory in the region of the highest barrier between the denatured and intermediate states. The differences between the two profiles at low values of natural coordinate (in the basins representing the native and intermediate states) result from the overlap between these basins on the natural coordinate. A better test of agreement is to compare the two SEKNs (Figure 3). The agreement between the two is good: the same five states are identified, with the same major folding pathways. Along these pathways the same rates are measured, within statistical error. Larger differences in rates are observed for transitions characterised by small rates (e. g. **i1** $\rightarrow$ **n2**) due to limited sampling of these routes. However, these rates have a negligible effect on the overall kinetics of the system.

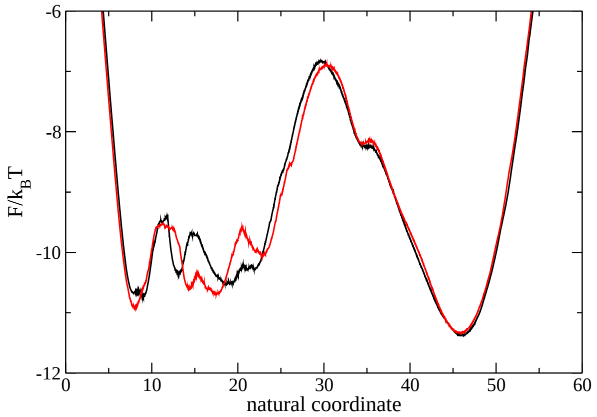

Figure 2: FEPs for the two halves of the trajectory of the wild-type λ repressor.

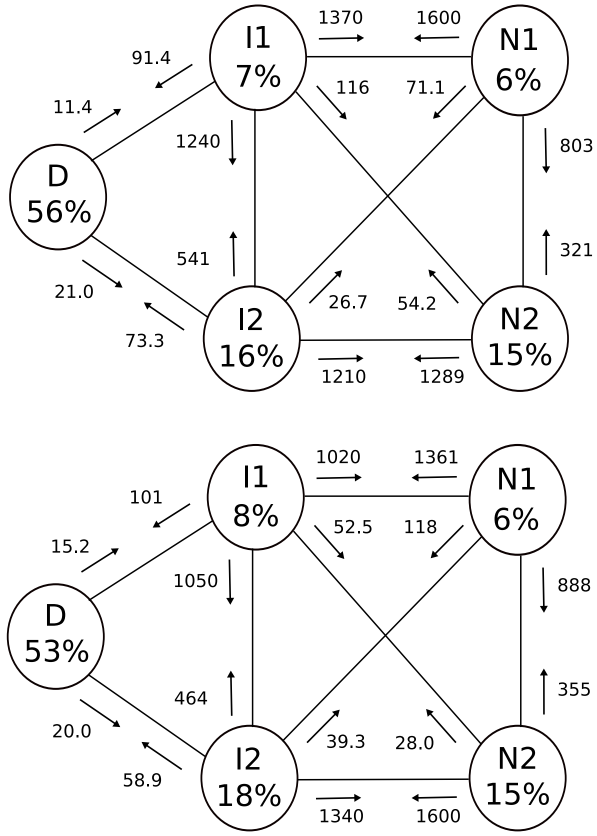

Figure 3: SEKNs for the two halves of the trajectory of the wild-type λ repressor.

References

[1] Krivov, S. V & Karplus, M. (2002) Free energy disconnectivity graphs: Application to peptide models. *J. Chem. Phys.* **117**, 10894–10903.

[2] Krivov, S. V & Karplus, M. (2004) Hidden complexity of free energy surfaces for peptide (protein) folding. *Proc. Natl. Acad. Sci. USA* **101**, 14766–14770.

[3] Hori, N, Chikenji, G, Berry, R. S, & Takada, S. (2009) Folding energy landscape and network dynamics of small globular proteins. *Proc. Natl. Acad. Sci. USA* **106**, 73–78.

[4] Krivov, S. V & Karplus, M. (2006) One-dimensional free-energy profiles of complex systems: Progress variables that preserve the barriers. *J. Phys. Chem. B* **110**, 12689–12698.

[5] Krivov, S. V, Muff, S, Caflisch, A, & Karplus, M. (2008) One-dimensional barrier preserving free energy projections of a β-sheet miniprotein: New insights into the folding process. *J. Phys. Chem. B* **112**, 8701–8714.

[6] Du, R, Pande, V. S, Grosberg, A. Y, Tanaka, T, & Shakhnovich, E. I. (1998) On the transition coordinate for protein folding. *J. Chem. Phys.* **108**, 334–350.

[7] Krivov, S. V & Karplus, M. (2008) Diffusive reaction dynamics on invariant free energy profiles. *Proc. Natl. Acad. Sci. USA* **105**, 13841–13846.
